# Low vitamin K status is a potential risk factor for COVID-19 infected patients: a systematic review and meta-analysis

**DOI:** 10.3389/fnut.2025.1476622

**Published:** 2025-04-07

**Authors:** Wei Liu, Xin Liu, Shiwei Kang, Yadong Yuan

**Affiliations:** ^1^First Department of Pulmonary and Critical Care Medicine, Hebei Chest Hospital, Shijiazhuang, China; ^2^Second Department of Pulmonary and Critical Care Medicine, The Second Hospital of Hebei Medical University, Shijiazhuang, China

**Keywords:** COVID-19, low vitamin K status, dephosphorylated-uncarboxylated matrix Gla protein, meta-analysis, dp-ucMGP

## Abstract

**Objective:**

To provide further data support for the treatment of COVID-19 by conducting a comprehensive analysis of reports on dephosphorylated-uncarboxylated Matrix Gla Protein (dp-ucMGP), which detects the functional vitamin K status post COVID-19 infection, using meta-analysis.

**Methods:**

This study conducted a comprehensive review and analysis of relevant research on dp-ucMGP detection in patients infected with COVID-19 through meta-analysis. The article collection period ranged from January 2024 to April 2024.

**Results:**

A total of 6 articles were included in this study. Baseline data analysis showed that the age of patients in the COVID-19 infected group was greater than that of the non-infected control group (*p* = 0.030); similarly, the age of patients in the severe infection group was also greater than that of the mild infection group (*p* = 0.003). In the analysis of underlying diseases, statistical differences were found between the Severe group and Mild group in the presence of CVD (*p* = 0.010). A total of 5 studies conducted dp-ucMGP detection in both the COVID-19 infected group and the control group. The results showed that the expression of dp-ucMGP was higher in the infected group than in the control group (*p* < 0.001). Subgroup analysis revealed that the expression of dp-ucMGP in the severe infection group was also higher than that in the mild infection group (*p* < 0.001).

**Conclusion:**

COVID-19 infected patients exhibit Low Vitamin K Status, which correlates positively with the severity of infection. Supplementation of vitamin K during COVID-19 infection may potentially mitigate the progression toward severe infection, necessitating further support from clinical data.

## 1 Introduction

Research shows that compared to healthy individuals, patients who are admitted to the Intensive Care Unit (ICU) or die after contracting COVID-19 have lower levels of vitamin K (1, 2). This may be due to COVID-19 infection causing inflammation in the lungs, damaging elastic fibers involved in lung respiration, leading to pulmonary fibrosis and thrombus formation (3). Vitamin K is crucial in activating protective proteins for elastic alveolar tissue in the lungs and participating in coagulation (4). Additionally, vitamin K is involved in the oxidative-reductive reactions of mitochondria (5). It can block the transmission of signals in infected cell mitochondria, preventing abnormal cell respiration infected by viruses, leading to their apoptosis.

Vitamin K is not a single nutrient but a group of compounds with similar structures. It mainly includes four forms: K1, K2, K3, and K4 (Supplementary Figure 1) (6). Among them, K1 and K2 are naturally synthesized and are fat-soluble vitamins, while K3 and K4 are synthetically produced and water-soluble (7). The status of vitamin K in the body can typically be assessed in two different ways: (1) by directly measuring the concentration of vitamin K in plasma and (2) by determining the amount of undercarboxylated vitamin K-dependent proteins. The first method can reflect recent vitamin K intake but is sensitive to triglyceride concentration and provides limited information on the utilization of vitamin K in tissues. Therefore, measuring the level of inactive (undercarboxylated) matrix Gla protein (MGP) in the blood can serve as a good biomarker for the functional vitamin K status in peripheral tissues (8). Vitamin K catalyzes the carboxylation of hepatic procoagulant and anticoagulant factors (9). It converts glutamic acid to *γ*-carboxy glutamic acid (Gla) residues, serving as an activator for hepatic factors II (prothrombin), VII, IX, and X, and also plays a pivotal role in the extracellular activation of protein S (10). MGP is a vitamin K-dependent inhibitor of soft tissue calcification and elastic fiber degradation (11). Insufficient or deficient extracellular vitamin K leads to a significant increase in inactive MGP, which is associated with the failure of vitamin K-mediated MGP carboxylation. Insufficient activation of vitamin K-dependent MGP renders elastic fibers unable to resist protein hydrolysis induced by SARS-CoV-2. Ultimately, the mechanism of vitamin K depletion induced by pneumonia leads to a reduction in activated MGP and protein S, exacerbating lung damage and coagulation dysfunction (12). Consequently, the dephosphorylated-uncarboxylated subtype of MGP (dp-ucMGP) reflects the functional vitamin K status and is currently considered the gold standard for measuring the vitamin K status in peripheral tissues (13). Typically, high levels of dp-ucMGP expression indicate severe deficiency of vitamin K in COVID-19 infected patients (1).

This study aims to compile and analyze data from relevant research on dp-ucMGP detection in COVID-19 infection. The goal is to summarize and elucidate the relationship between COVID-19 virus infection and vitamin K status in a quantitative manner. By doing so, we aim to provide more data references for understanding the pathogenesis and treatment of COVID-19 infection.

## 2 Materials and methods

### 2.1 Literature search

According to the established inclusion criteria, we collected and organized all articles meeting the inclusion requirements from January 2020 to April 2024. Keyword searches were conducted across databases including Google Scholar, PubMed, Cochrane Library, EBSCO, Web of Science, ScienceDirect, and Wiley. The search terms comprised (“COVID-19” or “Corona Virus Disease 2019” or “COVID, 2019” or “Corona Virus Disease, 2019” or “SARS-CoV-2” or “Severe Acute Respiratory Syndrome Coronavirus 2”) and (“Vitamin K” or “Matrix Gla Protein” or “dephosphorylated-uncarboxylatediso-form of MGP” or “dp-ucMGP”). This study is a meta-analysis, categorized under secondary analysis, thus excluding ethical considerations and factors such as informed consent from patients. We followed the recommendations of the PRISMA 2020 statement, providing a detailed description of the study’s screening, selection, data extraction, and analysis processes to ensure transparency and completeness in reporting quality (14).

### 2.2 Inclusion and exclusion criteria

The inclusion criteria for this study are as follows: (1) Patients infected with COVID-19 with detection of dp-ucMGP in their blood; (2) The study provided detailed data on the expression of dp-ucMGP in the COVID-19 infection group and the control group, or the prognosis of both groups; (3) Studies categorizing COVID-19 patients into severe and mild groups, or including control groups with and without infection.

Exclusion criteria: (1) Studies that only detect vitamin K without providing dp-ucMGP detection; (2) Studies providing dp-ucMGP detection results without other grouping criteria; (3) Studies with duplicated patient data; (4) Studies without quantifiable data; (5) Animal and cell-based studies.

### 2.3 Main testing indicators

The methods used for dp-ucMGP detection in each study are presented in Table 1. We collected, organized, and analyzed the expression levels of dp-ucMGP (pmol/L) in both the COVID-19 first-time infection group and the control group. Subgroup analysis was conducted to categorize patients within the COVID-19 infected group based on the severity of symptoms (including mortality). Baseline data such as age, gender, BMI (kg/m^2^), and underlying diseases including diabetes, hypertension, cardiovascular diseases, and lung diseases (such as Asthma/COPD) were collected for each subgroup and group. We assessed the risk of severe outcomes in COVID-19 infected patients associated with increased dp-ucMGP concentrations using odds ratios (OR) with corresponding lower limits (LL) and upper limits (UL).

**Table 1:** Basic information on included studies.

Author	Year	Region	Assays for dp-ucMGP	Type	Time	C-19	C
1. Linneberg A.	2020	Denmark	IDS-iSYS InaKtif MGP assay (Immunodiagnostic systems, plc, Tyne and Wear, UK)	Cohort	2020.03.10–2020.04.23	138	140
2. Anton S. M. Dofferhoff	2021	Netherlands	Commercially available IVD chemiluminescent InaKif MGP assay on the IDS-iSYS system (IDS, Boldon, United Kingdom)	Cohort	2020.03.12–2020.04.15	135	184
3. Margot P. J. Visser	2022	Netherlands	InaKtif MGP iSYS pre-commercial kit developed by IDS and VitaK	Cohort	2020	18	109
4. Ankita P. Desai	2021	USA	Not explicitly provided	Cohort	2020.04.21–2020.05.05	100	50
5. Mark M. G. Mulder	2023	Dutch	InaKif MGP assay on the IDS-iSYS system (IDS, Boldon, United Kingdom)	Cohort	2020.03.25–2021.04.13	112	NA
6. Huda M.	2023	Egypt	Not explicitly provided	Cohort	2021.10–2022.01	88	28

C-19, COVID-19 group; C, Control group.

### 2.4 Statistical methods

STATA 12.0 software was used to analyze and visualize the compiled data. Forest plots were employed to illustrate the calculated results. I^2^ was used to assess the heterogeneity of the studies. Due to the presence of high heterogeneity in the data, a random-effects model was adopted. For different types of variables, binary variables (n) and continuous variables (n, mean, and SD) were handled differently in the process of creating forest plots. Funnel plots were utilized to evaluate publication bias in included studies reporting categorical data, while Begg’s and Egger’s tests were employed to detect publication bias in studies reporting continuous data. A significance level of *p* < 0.05 was considered statistically significant for differences.

## 3 Results

### 3.1 Detailed inclusion process

Through keyword searches, a total of 547 relevant articles were initially considered for inclusion in this study. After reading and searching based on keywords and titles, 252 articles were excluded. Among the remaining 295 articles, further screening of abstracts and partial content led to the exclusion of 283 articles. From the remaining 12 articles, 6 were excluded due to being review articles, inability to extract data, or duplicate data. Ultimately, six articles were included in this study (Table 2).

**Table 2:** PRISMA 2020 flow diagram.

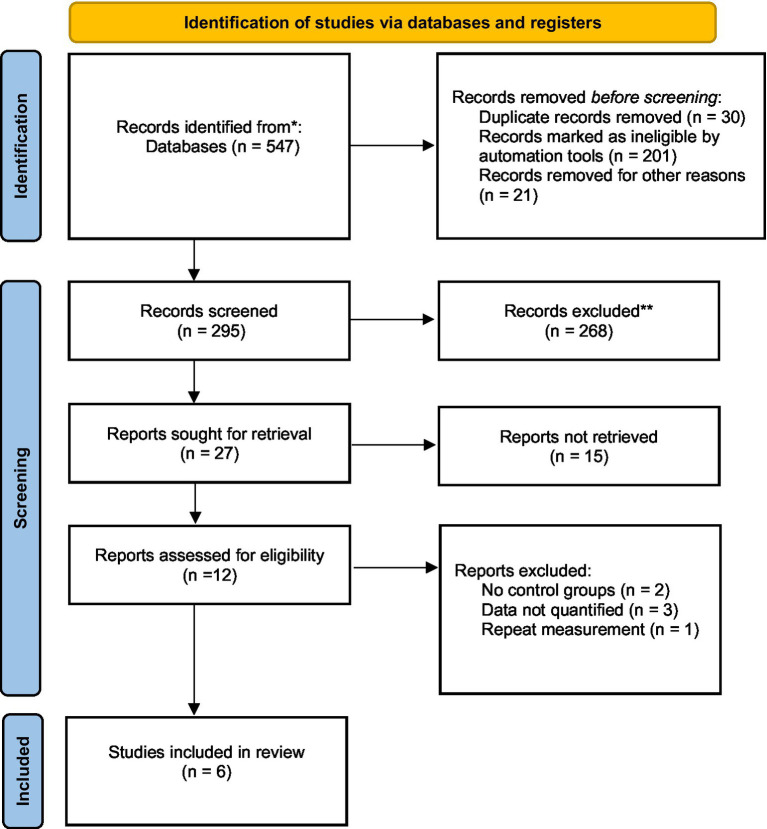

*Consider, if feasible to do so, reporting the number of records identified from each database or register searched (rather than the total number across all databases/registers).

**If automation tools were used, indicate how many records were excluded by a human and how many were excluded by automation tools (14).

### 3.2 Baseline parameters

**Figure 1:**
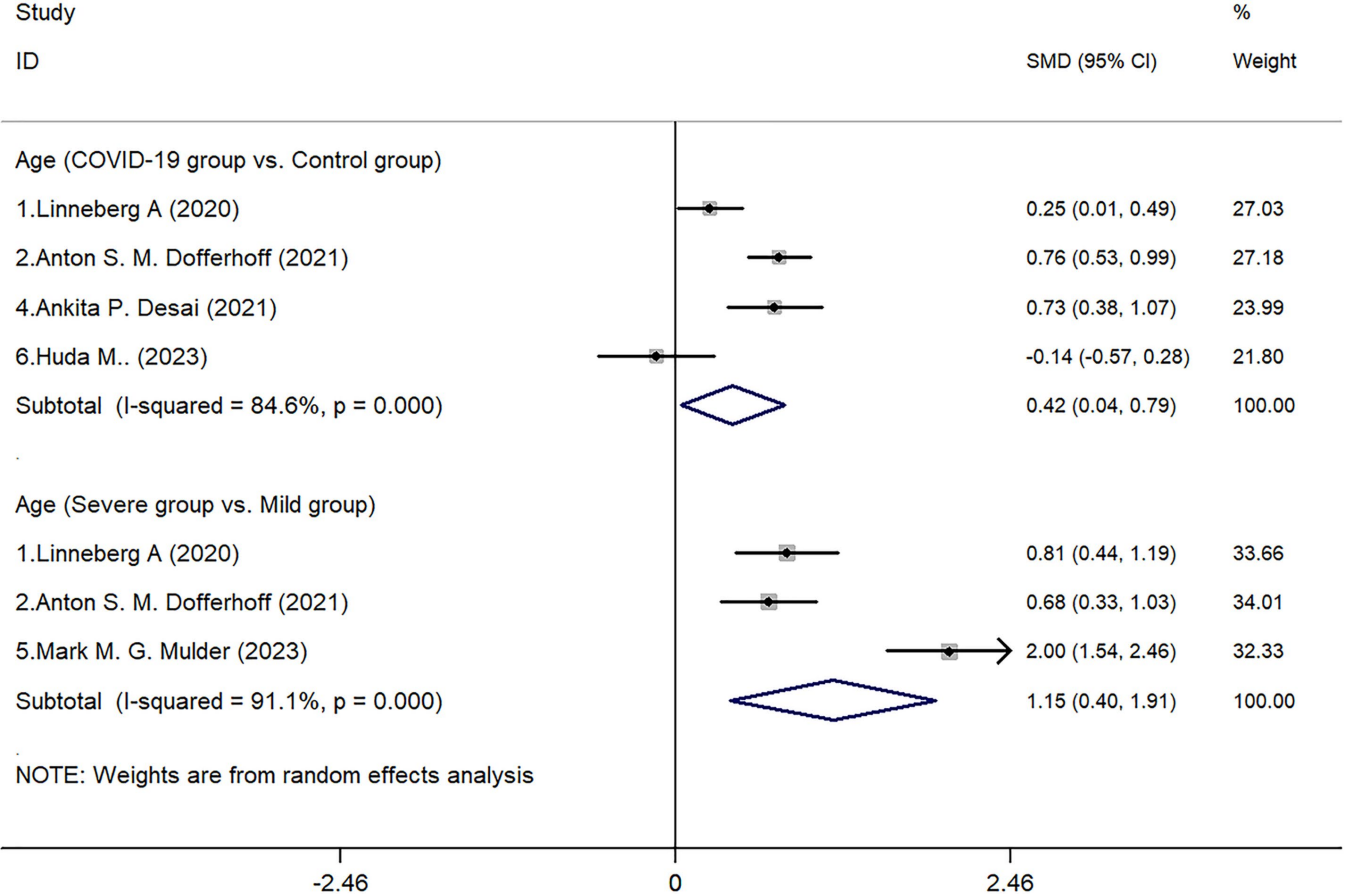
Forest map of age.

In all 6 included studies (1, 3, 15–18), 5 studies reported on both the COVID-19 infected group and the control group. The results showed that the age of patients in the COVID-19 infected group was higher than that of the control group (*Z* = 2.17, *p* = 0.030). Further subgroup analysis of age within the infected group showed a statistical difference between the severe group and the non-severe group (*Z* = 2.99, *p* = 0.003). Due to significant heterogeneity in the aforementioned analyses (I^2^ of 84.6 and 91.1% respectively), a random-effects model was utilized in drawing the forest plot (Figure 1).

**Figure 2:**
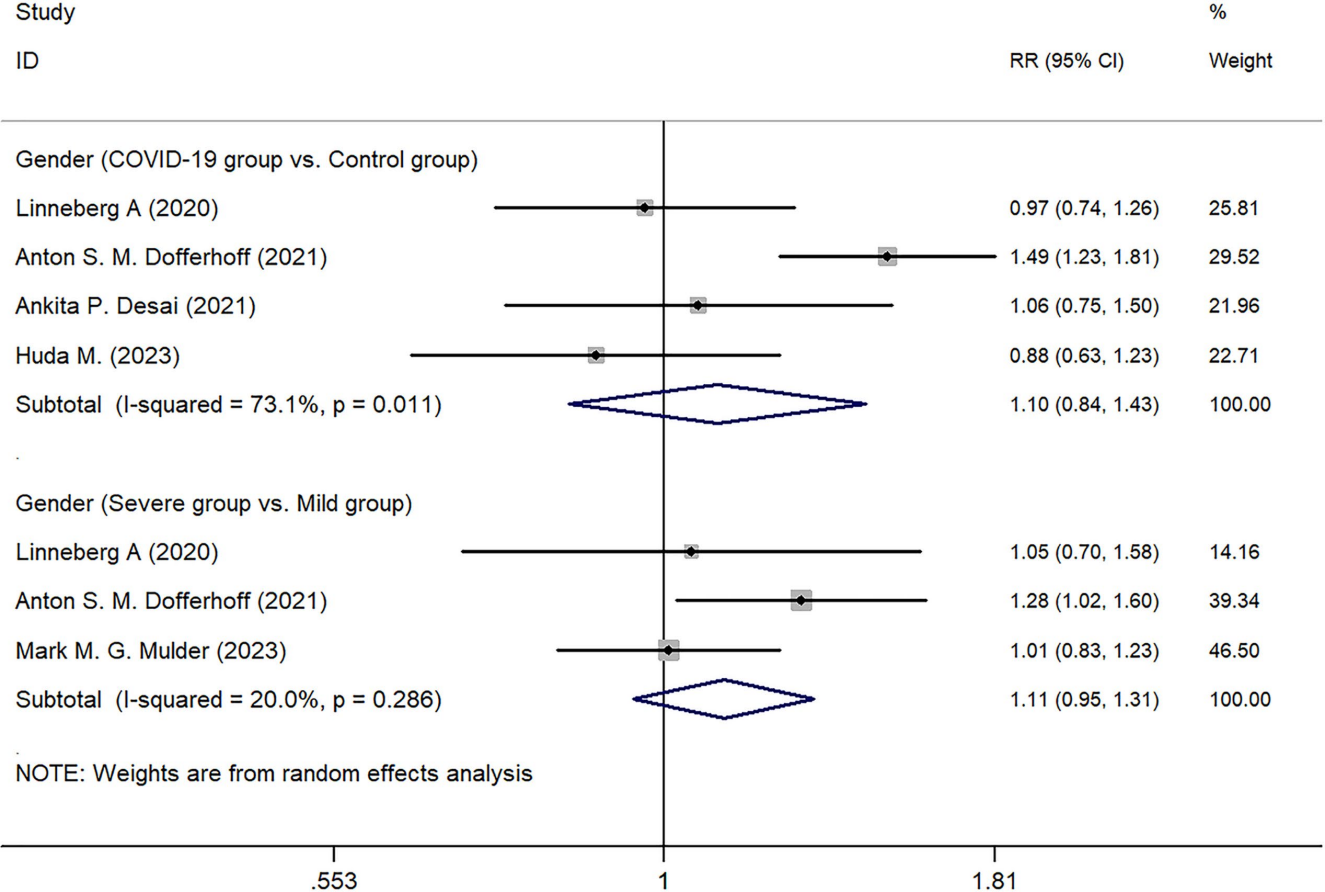
Forest map of gender.

Four studies conducted statistical analyses comparing patients in the COVID-19 infected group and the control group. The results showed that in the infected group, males accounted for 54.88% (253/461), while in the control group, males accounted for 47.02% (189/402). Further statistical analysis indicated no significant difference in gender distribution between the two groups (*Z* = 0.690, *p* = 0.489). Subgroup analysis within the COVID-19 infected group showed no statistical difference between the Severe group and the Mild group in terms of gender distribution (*Z* = 1.29, *p* = 0.197) (Figure 2).

**Figure 3:**
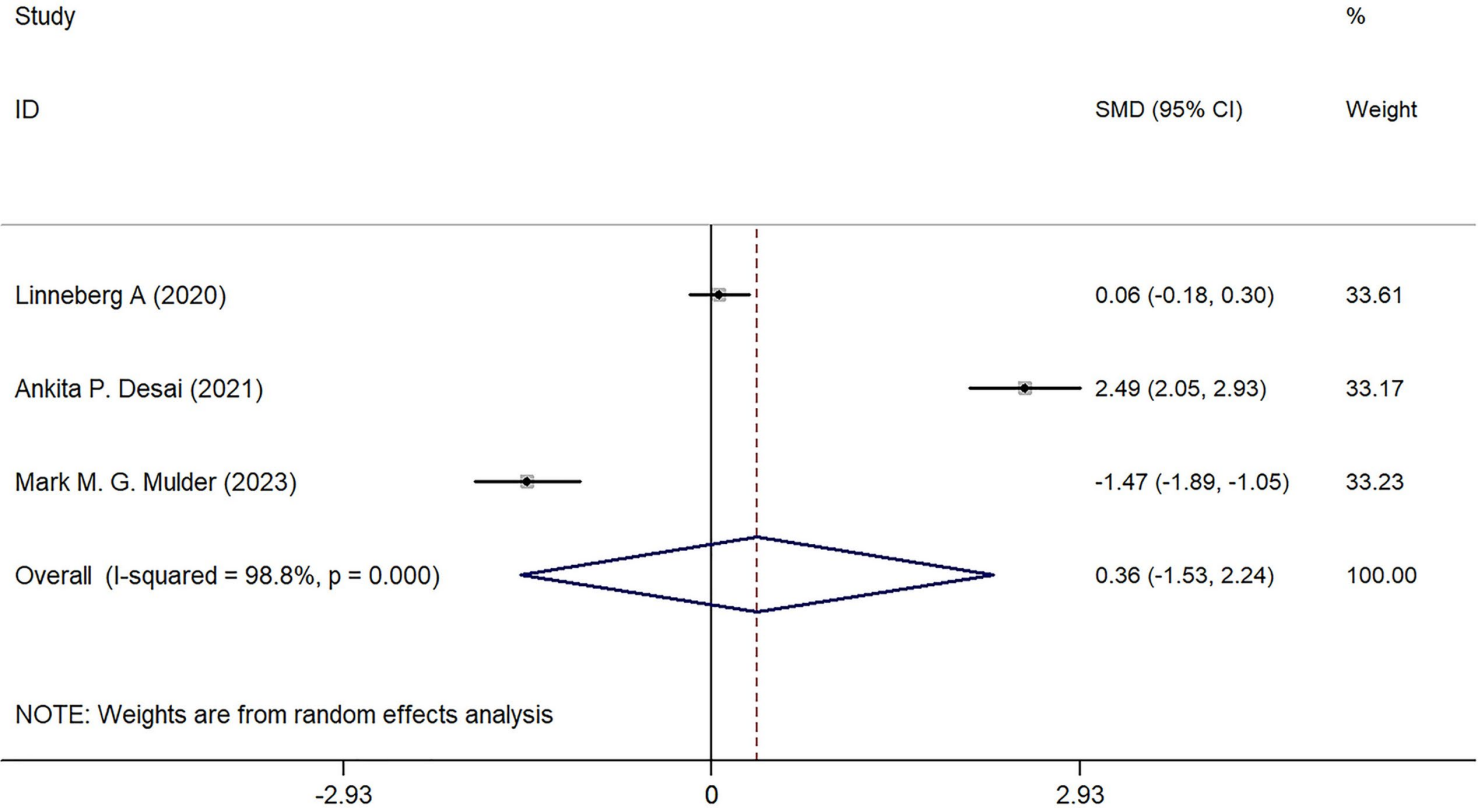
Forest map of BMI (kg/m^2^).

Three studies analyzed the BMI of patients in the COVID-19 infected group, but no statistical difference was found in the comparison between the Severe group and the Mild group (*Z* = 0.370, *p* = 0.709) (Figure 3).

### 3.3 Underlying disease

**Figure 4:**
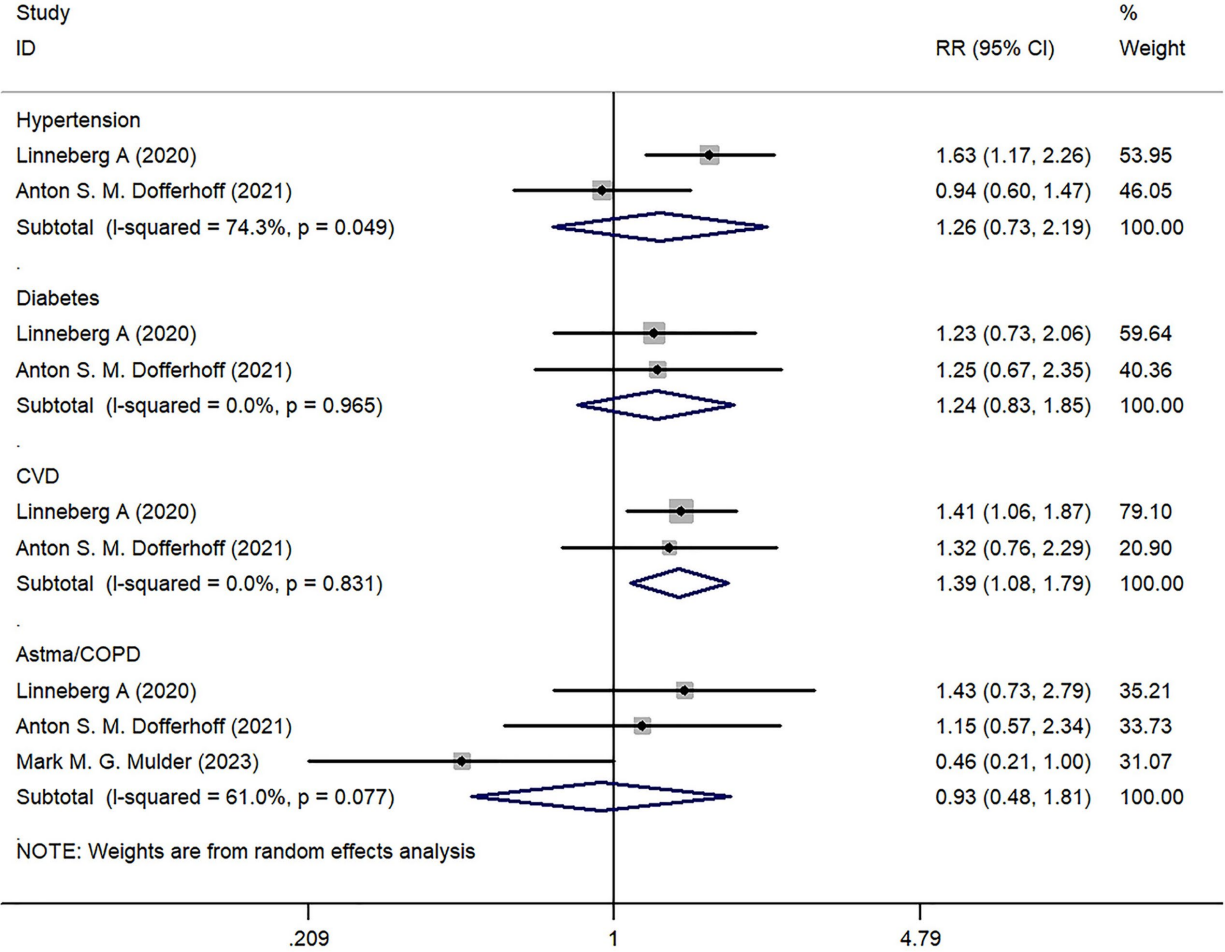
Forest map of underlying diseases.

We analyzed the underlying diseases of COVID-19 infected patients included in the study. Two studies reported on the prevalence of hypertension, diabetes, and cardiovascular disease (CVD). There was no statistically significant difference in hypertension and diabetes between the Severe group and the Mild group (*p* > 0.05); however, there was a statistical difference in CVD between the two groups (*Z* = 2.57, *p* = 0.010) (Figure 4).

Three studies reported on the incidence of Asthma/COPD, but there was no statistically significant difference in comparison between the two groups (*Z* = 0.20, *p* = 0.838) (Figure 4).

### 3.4 Expression of dp-ucMGP

**Figure 5:**
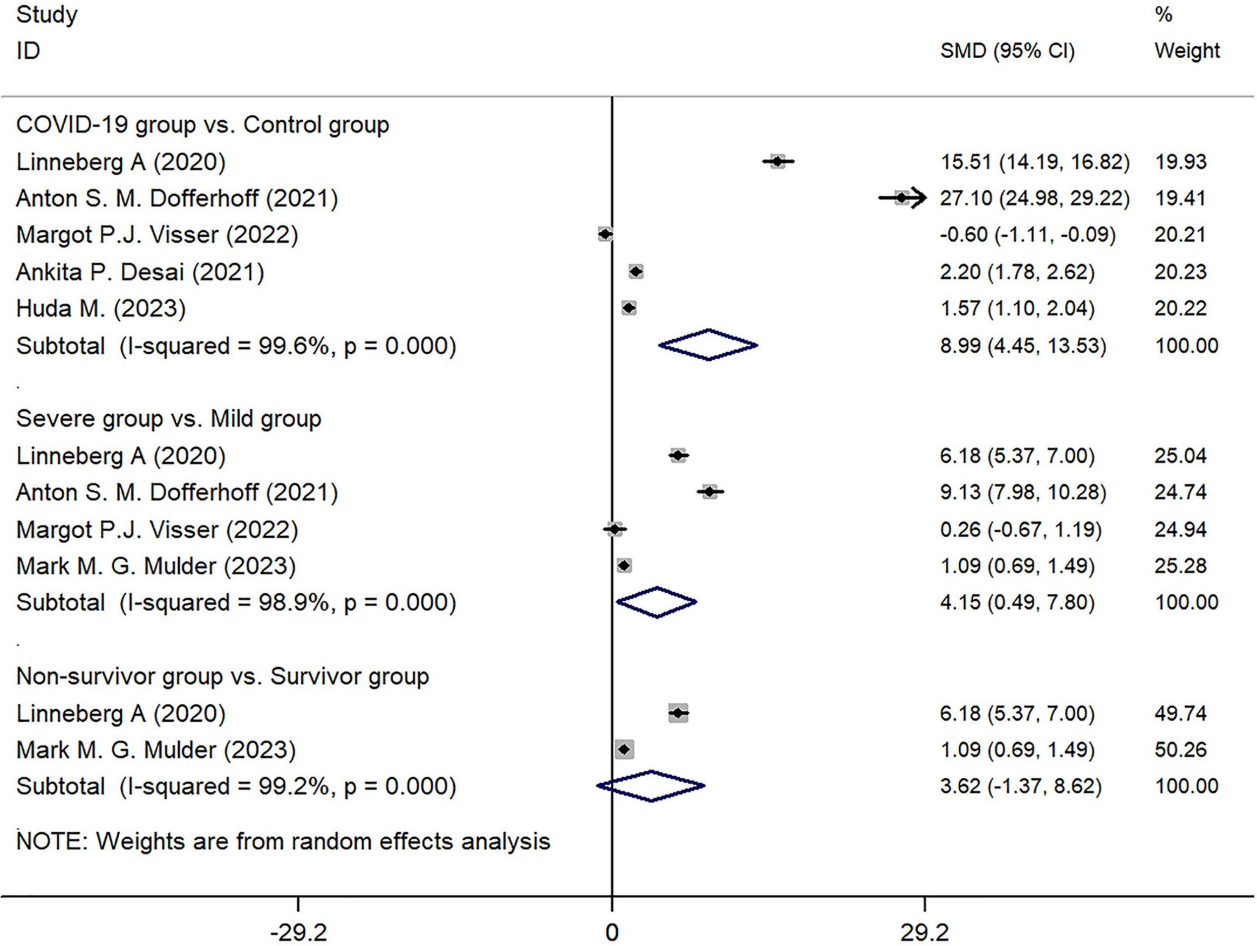
Forest map of dp-ucMGP.

Among the 6 studies included, 5 studies reported on the expression of dp-ucMGP in the COVID-19 infected group (*n* = 479) and the control group (*n* = 506). After collecting and organizing the data from these studies and performing the necessary transformations, the results showed that the expression of dp-ucMGP was higher in the infected group compared to the control group, and there was a statistically significant difference between the two groups (*Z* = 16.32, *p* < 0.001) (Figure 5).

Four studies categorized the infected group into mild and severe based on the severity of COVID-19 infection. The comparison between these two subgroups revealed that the expression of dp-ucMGP was higher in the severe group compared to the mild group, and the difference was statistically significant (*Z* = 2.22, *p* < 0.026). However, when grouping based on mortality, although there was a trend for higher expression of dp-ucMGP in the death group compared to the non-death group, the difference between the two groups was not statistically significant (*Z* = 1.42, *p* = 0.155). Due to the high heterogeneity observed in these subgroup analyses (I^2^ of 99.6, 98.9, and 99.2% respectively), a random-effects model was used to generate forest plots. Detailed results can be found in Figure 5.

### 3.5 Publication bias

**Figure 6:**
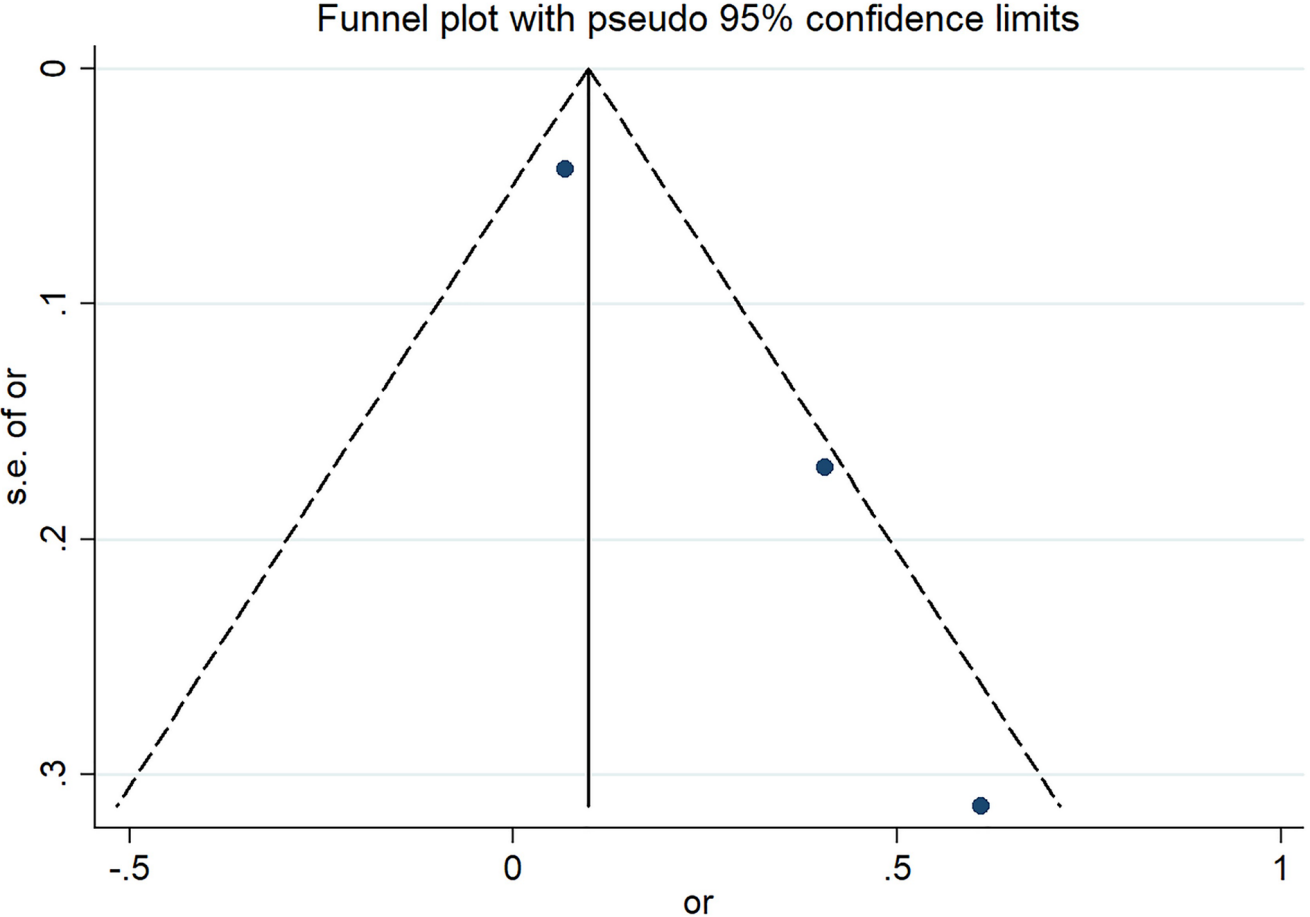
Funnel plot.

The funnel plot presents the assessment results of the risk of severe outcomes in COVID-19 infected patients reported in three studies. The results indicate that there is no significant publication bias in these three studies (Figure 6).

## 4 Discussion

This study conducted a quantitative summary analysis using meta-analysis methods on six studies that met the inclusion criteria and involved the expression of dp-ucMGP in COVID-19 patients. The results indicate a significant depletion of vitamin K following COVID-19 infection, and the extent of vitamin K depletion is correlated with the severity of infection, as reflected in the analysis of dp-ucMGP concentrations. Analysis of baseline data revealed that the age of patients in the COVID-19 infected group was higher than that in the non-infected control group (*p* = 0.030), and the age of patients in the severe infection group was also higher than that in the mild infection group (*p* = 0.003). These findings are consistent with the research by Liu et al. (19), which suggests that aging is associated with physiological, pathological, and functional changes in the lungs. Based on the results, gender does not appear to be a significant factor in COVID-19 infection or disease severity. Although there was a slightly higher proportion of males in the infected group (54.88%) compared to the control group (47.02%), the statistical analysis showed no significant difference (*p* = 0.489). Similarly, within the infected group, there was no significant difference in gender distribution between severe and mild cases (*p* = 0.197). These findings suggest that gender may not be a determining factor in susceptibility to COVID-19 or in disease progression. According to a report by the World Health Organization, the distribution of COVID-19 infections between males (51%) and females (47%) is relatively balanced (20). However, the mortality rate is significantly higher in males (58%), with a statistically significant difference between sexes (*p* < 0.05). This finding is supported by a multicenter retrospective study conducted by Wuhan University, which indicated that although the number of infections is comparable, male patients tend to experience more severe disease progression (21). In contrast, females exhibit a more extended incubation period. Therefore, this study suggests that while sex may not be a decisive factor in COVID-19 susceptibility, further research is warranted to elucidate the underlying biological and social determinants contributing to sex differences in disease progression and clinical outcomes.

Regarding BMI, three studies analyzed its potential impact on disease severity. However, the comparison between the Severe and Mild groups showed no statistically significant difference (*p* = 0.709). Based on the available data, this indicates that BMI may not play a crucial role in determining COVID-19 severity. While some studies have suggested potential associations between gender, BMI, and COVID-19 outcomes, the current findings do not support a strong correlation. For instance, a study employing the Mendelian randomization method demonstrated that smoking and obesity increase the risk of severe COVID-19 by 65–81% (22). Their findings indicated that for each standard deviation increase in BMI, the risk of developing severe COVID-19 rises by 81%, the risk of hospitalization by 55%, and the risk of infection by 18%. Additionally, a study conducted by the U.S. Centers for Disease Control and Prevention (CDC) identified a nonlinear association between BMI and COVID-19 severity, revealing that the lowest risk occurs within the healthy weight (18.5–24.9) and overweight (25–29.9) ranges (23). At the same time, a progressive increase in BMI corresponds to a significant escalation in risk. Collectively, these findings suggest that while moderate overweight may not substantially exacerbate COVID-19 severity, both underweight and obesity may contribute to heightened risk. It is posited that BMI may not serve as the primary determinant of COVID-19 severity; however, extreme deviations in body weight could be associated with adverse outcomes. Further large-scale investigations are warranted to substantiate these conclusions.

In terms of underlying diseases, the results show no statistical difference between the Severe group and the Mild group in terms of hypertension, diabetes, and lung-related diseases (including Asthma/COPD), except for CVD. Despite COVID-19 being primarily a respiratory system disease, it can affect the cardiovascular system through various mechanisms. Studies have shown that pre-existing cardiovascular diseases and cardiovascular risk factors increase susceptibility to COVID-19 infection. Furthermore, COVID-19 can exacerbate underlying CVD and even precipitate the occurrence of new cardiac complications (24). Therefore, clinicians should appropriately manage CVD patients with COVID-19 infection and monitor the acute cardiac condition of patients to prevent death and critical conditions (25).

When the novel coronavirus enters the alveolar cells, it can cause infection in the pulmonary epithelial and endothelial cells, damaging the respiratory tract (26). The infection triggers the excessive production of pro-inflammatory cytokines (such as IL-6, TNF-a, IL-1, and CRP), thereby inducing acute respiratory distress syndrome (27). This further leads to an elevation in matrix metalloproteinases (MMPs) 8 and 9 levels, resulting in pulmonary fibrosis (28). Simultaneously, it affects the normal clotting process, leading to abnormalities in blood coagulation (including intravascular coagulation disorders, thrombus formation, and pulmonary microthrombi) (29). These pathogenic mechanisms are associated with insufficient carboxylation of matrix Gla protein (MGP), protein S, and protein C, and all of the above processes require the synergistic involvement of vitamin K. Research has found severe extracellular vitamin K deficiency in patients infected with Covid-19 (12).

Research on vitamin K deficiency in COVID-19 patients suggests that developing corresponding preventive and treatment strategies could benefit infected individuals. Mangge et al. (30) conducted a study involving 77 Covid-19 patients, suggesting that supplementing vitamin K2 could be a cost-effective and effective method for preventing severe Covid-19 progression. DaeiSorkhabi et al., through a review, further argue the biological and clinical rationale for vitamin K supplementation as a potential adjunctive therapy for COVID-19 (31). Preliminary research by Nuszkiewicz et al. (32) suggests that vitamin K may reduce lipid peroxidation and inhibit ferroptosis, contributing to the treatment of COVID-19 patients. They suggest that supplementing vitamin K during COVID-19 infection may positively impact the severity of the disease in infected patients. Rivera-Caravaca et al. (33) observation on the outpatient treatment of COVID-19-infected patients with oral anticoagulants and vitamin K antagonists (Warfarin) showed that despite similar risks and 30-day event-free survival rates in terms of all-cause mortality, ICU admission necessity and gastrointestinal bleeding between patients receiving VKA and those receiving DOAC treatment; the risk of any arterial or venous thrombotic event in the VKA cohort was 43% higher (OR 1.43, 95% CI 1.03–1.98, *p* = 0.029). This study suggests caution in using vitamin K antagonists in anticoagulant therapy for COVID-19 infection, underscoring the close association between excessive vitamin K depletion after COVID-19 infection and the progression of pneumonia in patients. Desai et al. (1) study further corroborated this viewpoint. Their research showed that excessive vitamin K status (dp-ucMGP) and D depletion was independently associated with the worsening severity of COVID-19 pneumonia. Visser et al. (34) also showed that COVID-19 pneumonia patients who received vitamin D supplementation after correcting vitamin K deficiency could benefit from the positive effects of vitamin D, thus avoiding excessive lung damage caused by elastic fiber calcification. This research direction provides further prospects for applying vitamin K in treating COVID-19 infection. In a single-center, phase 2, double-blind, randomized, placebo-controlled trial conducted in 2024 by Visser et al., hospitalized COVID-19 patients who received daily supplementation of 999 mcg of vitamin K2 (menaquinone-7, MK-7) exhibited good tolerance with no increase in adverse events (35). A linear mixed model analysis showed that the levels of dp-ucMGP and PIVKA-II in the supplementation group were significantly reduced compared to the control group (*p* = 0.008 and *p* = 0.0017, respectively). Their study demonstrated that vitamin K2 supplementation in hospitalized COVID-19 patients is safe and significantly improves vitamin K status by reducing dp-ucMGP levels.

This study has certain limitations. For instance, as a meta-analysis, we could not access the original data from the included studies, which constrained further in-depth analyses and validation. The conclusions of a meta-analysis rely on the quality and availability of data from published studies; thus, potential biases or limitations in the original studies may affect the reliability of our findings. Additionally, due to the limited number of included studies, we could not perform more detailed subgroup analyses to evaluate the relationship between vitamin K levels and COVID-19 risk in different populations. Although our research suggests that low vitamin K levels may be a potential risk factor for COVID-19, it does not specify which specific populations this conclusion applies to, limiting its clinical applicability.

## 5 Conclusion

This study systematically evaluated the detection of dp-ucMGP in COVID-19-infected patients through a meta-analysis, aiming to provide more detailed clinical evidence for the prevention and treatment strategies involving vitamin K during the COVID-19 pandemic. The results indicate that COVID-19-infected patients generally exhibit low vitamin K status, which is significantly correlated with the severity of infection. Therefore, supplementation with vitamin K may offer benefits in slowing the progression of the disease to severe stages. However, given the preliminary nature of these findings, further clinical research is required to validate the actual effects and safety of vitamin K supplementation in COVID-19 patients. Considering the individual variability and pathological complexity of COVID-19 patients, future studies should comprehensively account for multiple factors, such as risk factors, age, viral strains, and vaccination status, and perform more thorough and systematic analyses.

## Supplementary material

The Supplementary material for this article can be found online at: https://www.frontiersin.org/articles/10.3389/fnut.2025.1476622/full#supplementary-material

Supplementary Figure 1 The four chemical structures of vitamin K.
