# Supplementary figures and images for: Low vitamin K status is a potential risk factor for COVID-19 infected patients: a systematic review and meta-analysis
Source: Front Nutr. 2025 Apr 7;12:1476622. doi: 10.3389/fnut.2025.1476622 (PMC12009694; doi:10.3389/fnut.2025.1476622)

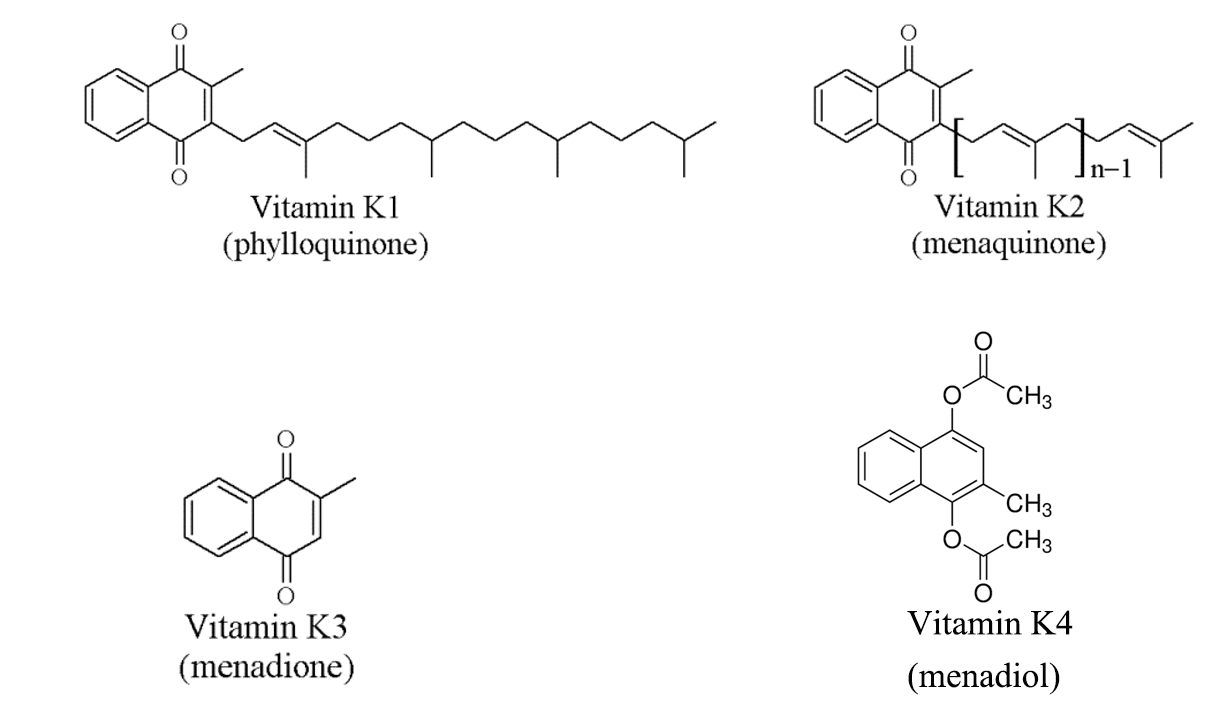

Supplement: Supplementary Figure 1 — The four chemical structures of vitamin K. [file Image_1.tif]
